# *MoLAEA* Regulates Secondary Metabolism in Magnaporthe oryzae

**DOI:** 10.1128/mSphere.00936-19

**Published:** 2020-04-01

**Authors:** Pallabi Saha, Suvranil Ghosh, Subhankar Roy-Barman

**Affiliations:** a Department of Biotechnology, National Institute of Technology, Durgapur, India; b Division of Molecular Medicine, Bose Institute, Kolkata, India; University of Texas Health Science Center

**Keywords:** sporulation, melanin, pathogenicity, penicillin G, secondary metabolism, velvet family of proteins

## Abstract

Fungi are rich sources of secondary metabolites of pharmaceutical importance, such as antibiotics, antitumor agents, and immunosuppressants, as well as of harmful toxins. Secondary metabolites play important roles in the development and pathogenesis of fungi. LaeA is a global regulator of secondary metabolism and was originally reported in Aspergillus nidulans; however, its role in secondary metabolism in Magnaporthe oryzae has not yet been reported. Here, we investigated the role of a gene homologous to *LAEA* (loss of *AflR* expression) of *Aspergillus* spp. in Magnaporthe oryzae, named M. oryzae *LAEA* (*MoLAEA*). Studies on *MoLAEA* overexpression and knockdown strains have suggested that this gene acts as a negative regulator of sporulation and melanin synthesis. However, it is not involved in the growth and pathogenesis of M. oryzae. Transcriptomic data indicated that *MoLAEA* regulated genes involved in secondary metabolism. Interestingly, we observed (for the first time, to our knowledge) that this gene is involved in benzylpenicillin (penicillin G) synthesis in M. oryzae. Overexpression of *MoLAEA* increased penicillin G production, whereas the silenced strain showed a complete absence of penicillin G compared to its presence in the wild type. We also observed that *Mo*LaeA interacted with *Mo*VeA, a velvet family protein involved in fungal development and secondary metabolism, in the nucleus. This study showed that though *MoLAEA* may not make any contribution in rice blast fungal pathogenesis, it regulates secondary metabolism in M. oryzae and thus can be further studied for identifying other new uncharacterized metabolites in this fungus.

**IMPORTANCE** M. oryzae causes blast disease, the most serious disease of cultivated rice affecting global rice production. The genome of M. oryzae has been shown to have a number of genes involved in secondary metabolism, but most of them are uncharacterized. In fact, compared to studies of other filamentous fungi, hardly any work has been done on secondary metabolism in M. oryzae. It is shown here (for the first time, to our knowledge) that penicillin G is being synthesized in M. oryzae and that *MoLAEA* is involved in this process. This is the first step in understanding the penicillin G biosynthesis pathway in M. oryzae. This study also unraveled the details of how *Mo*LaeA works by forming a nuclear complex with *Mo*VeA in M. oryzae, thus indicating functional conservation of such a gene across filamentous fungi. All these findings open up avenues for more relevant investigations on the genetic regulation of secondary metabolism in M. oryzae.

## Abstract (precis)

M. oryzae causes blast disease, the most serious disease of cultivated rice affecting global rice production. The genome of M. oryzae has been shown to have a number of genes involved in secondary metabolism, but most of them are uncharacterized. In fact, compared to studies of other filamentous fungi, hardly any work has been done on secondary metabolism in M. oryzae. It is shown here (for the first time, to our knowledge) that penicillin G is being synthesized in M. oryzae and that *MoLAEA* is involved in this process. This is the first step in understanding the penicillin G biosynthesis pathway in M. oryzae. This study also unraveled the details of how *Mo*LaeA works by forming a nuclear complex with *Mo*VeA in M. oryzae, thus indicating functional conservation of such a gene across filamentous fungi. All these findings open up avenues for more relevant investigations on the genetic regulation of secondary metabolism in M. oryzae.

## INTRODUCTION

Secondary metabolites are naturally occurring organic compounds produced by fungi, bacteria, and plants. Fungi are known to produce various bioactive secondary metabolites of pharmaceutical and agricultural importance, such as antibiotics, antitumor metabolites, pesticides, and insecticides, as well as deleterious mycotoxins and cytotoxic carcinogenic compounds (1). These medically and industrially important compounds have boosted the production of and research on fungal secondary metabolites (2). Secondary metabolite production starts late in the growth phase of an organism, i.e., during the stationary or resting phase (3). In contrast to the genes involved in primary metabolism, secondary-metabolism-associated genes are found clustered in the fungal genome (4) and hence can be easily identified in the genome. Unlike primary metabolites, secondary metabolites are not directly required for fungal growth (5). Secondary metabolites assist microorganisms in defending their habitat and in protecting them from competitors and are also required for communication (6, 7). Genes encoding polyketide synthases (PKS), nonribosomal peptide synthetases (NRPS), P450 monooxygenase, methyltransferase, and reductase are some of the structural genes involved in the synthesis of secondary metabolites (8, 9). Secondary metabolism has often been found to be associated with sporulation in fungi (3, 10). Metabolites such as linoleic acid, zearalenone, and butyrolactone I induce sporulation (11–13), whereas melanin protects the spores from UV irradiation and is required for virulence (14, 15).

*LAEA* (loss of *AflR* expression) was first reported in Aspergillus nidulans in complementation analysis of the sterigmatocystin mutant (16). The global regulation of secondary metabolism first came to light with the identification of LaeA in A. nidulans. LaeA is a novel methyltransferase whose S-adenosyl methionine (SAM) binding site is required for its function, and LaeA is known to automethylate a methionine residue near the SAM-binding site (17, 18). LaeA is a part of the velvet trimeric complex that is formed in the nucleus in dark and that consists of LaeA, VeA, and VelB (19–22). Reports show that VelB interacts with the N terminus of VeA and that LaeA interacts with the C terminus of VeA in A. nidulans (23).VeA, VelB, VelC, and VosA of the velvet family of proteins are involved in fungal development and secondary metabolism in A. nidulans (24). The velvet family of proteins regulates conidiation, infection-related development, and pathogenesis in Magnaporthe oryzae (25). LaeA not only forms trimeric complexes in A. nidulans but also is responsible for controlling expression, modifications, and interactions of the velvet proteins (22). Although 15 years have passed since its identification, LaeA remains an important protein in identifying cryptic secondary-metabolite clusters.

In A. nidulans, *LAEA* regulates the synthesis of lovastatin, sterigmatocystin, and penicillin (16). In Aspergillus fumigatus, it helps in the synthesis of gliotoxin, fumagillin, fumagatin, helvolic acid, and mycelial pigments (9). Overexpression of *LAEA* in Aspergillus flavus was previously shown to lead to the overproduction of several metabolites, including aflatoxin, cyclopiazonic acid, kojic acid, oryzaechlorin, and asperfuran (26). In Penicillium chrysogenum, *LAEA* plays an important role in penicillin production. Overexpression of *LAEA* in P. chrysogenum increased penicillin production by 25% (27, 28). In *Fusarium* spp., it is responsible for the synthesis of bikaverin, fumonisins, fusaric acid, and tricothecenes (29, 30). It regulates the synthesis of oxalic acid in *Botrytis cinerea* (31). *LAEA* not only acts as a positive regulator of secondary metabolism but has also been previously reported to negatively regulate the synthesis of dothistromin, a polyketide virulence factor in Dothistroma septosporum (32).

M. oryzae is responsible for blast disease worldwide and is one of the most dreaded plant pathogens, causing from 10% to 30% yield losses every year (33, 34). This annual loss in the production could feed 60 million people (35).The pathogen infects rice and other grasses by forming a dome-shaped infectious structure known as the appressorium that is formed from a two-to-three-celled germinating asexual spore attached to the plant tissue. The appressorium generates enough turgor pressure to rupture the plant cuticle and cause the infection (33, 36, 37). Analysis of the M. oryzae genome has revealed several genes encoding enzymes involved in secondary metabolism (38).Twenty-three genes have been predicted to encode PKS, six genes encode NRPS, and eight genes encode PKS-NRPS hybrids (38). Most of the genes involved in secondary metabolism in M. oryzae remain uncharacterized. Therefore, understanding the role of global regulators of secondary metabolites is critical as this will further assist in utilizing the silent metabolite clusters and identifying new metabolites.

In this report, we present a functional characterization of M. oryzae *LAEA* (*MoLAEA*) in M. oryzae, which is homologous to *LAEA* in *Aspergillus* spp. We developed overexpression and knockdown strains (which generally have opposing phenotypes) of *MoLAEA* after unsuccessful attempts to generate knockout mutants of this gene. This gene was not required for the morphological development or pathogenesis of M. oryzae. However, it negatively regulated sporulation as well as melanin biosynthesis. Furthermore, overexpression of *MoLAEA* resulted in increased penicillin G synthesis, whereas the silenced strain showed a complete absence of the same. This is an important observation, as M. oryzae has not been known to produce penicillin G to date. However, further efforts are required for investigating the penicillin biosynthesis pathway in M. oryzae. On the other hand, we were able to uncover the mechanism via which *Mo*LaeA functions in M. oryzae by forming a nuclear complex with members of the velvet family that includes protein *Mo*VeA, indicating conservation of its role across filamentous fungi. As *LAEA* is known as the global regulator of secondary metabolism, this report will help in identifying other new metabolites and relevant biosynthetic pathways in M. oryzae.

## RESULTS

### Identification of a *LAEA* homologue in M. oryzae.

BLASTP analysis of A. nidulans LaeA against the M. oryzae database (BroadMIT version 6) predicted the product of gene *MGG_07964* (1,812 bp long with a coding sequence of 1,020 bp) to be the best candidate gene. It was 38% homologous to the LaeA of A. nidulans, 39% similar to that of A. flavus, 38% similar to that of A. fumigatus, and 37% similar to that of A. niger. Therefore, we named this gene *MoLAEA*. *MoLAEA* is located on chromosome 3 of M. oryzae and encodes a protein consisting of 339 amino acids with a methyltransferase domain, a characteristic feature observed in LaeA studied in other filamentous fungi. Multiple-sequence alignment of *Mo*LaeA, including amino acid sequences of LaeA from *Aspergillus* spp., was carried out to evaluate the conservation of the methyltransferase domain (see Fig. S1 at https://figshare.com/authors/Subhankar_Roy-Barman/8596764).

### *MoLAEA* does not affect fungal growth.

To investigate the biological role of *MoLAEA*, the gene was both silenced and overexpressed in M. oryzae. These transformants were confirmed using Southern hybridization with *PtrpC* (promoter region in pSilent1 and pSilentDual1) as the probe. Two silencing transformants, one in pSilentDual1 named pSD1-2 and another in pSilent1 named pS4, and one overexpression transformant in pSilent1 named OE2 (overexpression 2) were selected for the study. Compared to the wild type, the overexpression transformant showed a 4.4-fold increase in expression of *MoLAEA*, whereas knockdown transformants pSD1-2 and pS4 showed 5-fold and 3.3-fold decreases, respectively, in expression of *MoLAEA*. All the transformants selected for functional characterization of *MoLAEA* harbored a single integration event (see Fig. S2 and S3).

**FIG 1:**
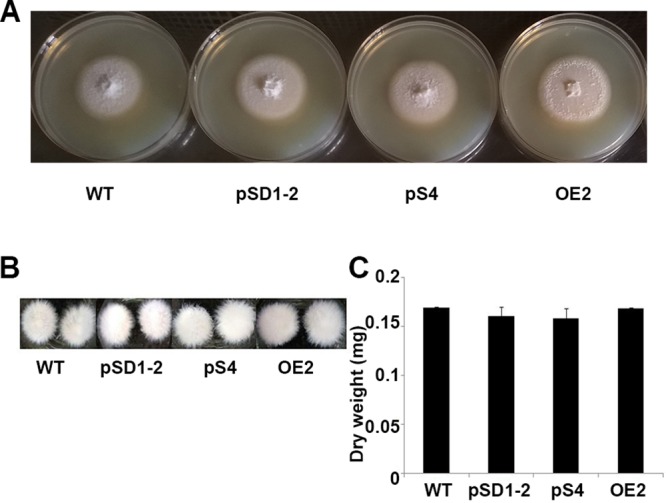
Effect of *MoLAEA* on the growth of M. oryzae. (A) The wild-type (WT), overexpression, and knockdown strains were grown on complete media at 28°C for 7 days. (B) The phenotype of the mycelial growth in liquid complete media for 48 h at 28°C. (C) Dry weight of the fungal biomass of the wild-type, overexpression, and knockdown strains incubated at 50°C for 72 h. The error bars represent standard deviations of results from triplicate measurements.

To determine whether *MoLAEA* was involved in growth, the growth rates of the wild-type, overexpression, and knockdown strains grown in complete media for 7 days were monitored (Fig. 1). The results showed that overexpression or silencing of *MoLAEA* did not affect the growth of the strains in solid or liquid media, although the level of aerial hyphal growth was lower in the overexpression strain than in the wild type.

We also checked the involvement of *MoLAEA* in maintaining the cell wall integrity of the fungus. There were no significant differences in the levels of radial growth of the overexpression and knockdown strains grown in media supplemented with Congo red or hydrogen peroxide or in the optical density with respect to peroxidase or laccase activity (data not shown). Thus, these findings indicated that *MoLAEA* neither has a role in growth of the fungus nor is involved in maintaining the cell wall integrity.

### *MoLAEA* negatively regulated melanin synthesis.

**FIG 2:**
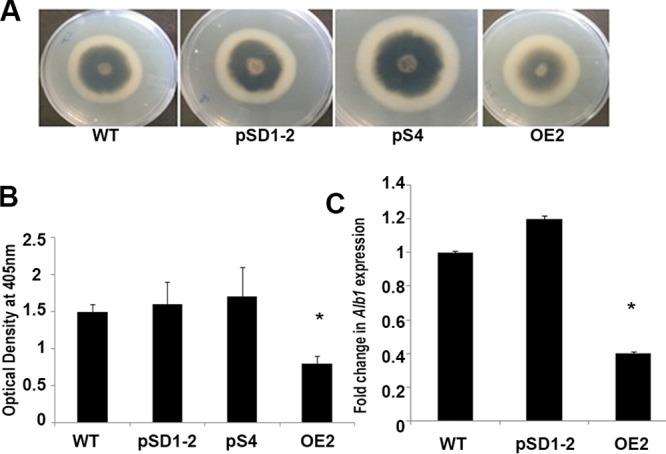
Effect of *MoLAEA* on melanin synthesis. (A) The wild-type, overexpression, and knockdown strains were inoculated on yeast extract-glucose (YEG) media and grown for 7 days at 28°C. (B) The amount of melanin produced was measured at 405 nm. (C) Expression of *Alb1* in the wild-type, overexpression, and knockdown strains. Error bars represent standard deviations, and asterisks represent significant differences between the wild-type and mutant strains at *P* < 0.05.

Melanin is a critical secondary metabolite that is crucial for generating turgor pressure for the appressoria to penetrate into host tissue. It also protects the spores from damage from UV light (39). We observed that the level of melanin content was visibly higher in the silenced strains and lower in the overexpression strain (Fig. 2A). To further confirm this observation, the optical densities of the supernatants of the crushed biomass of the wild-type, overexpression, and knockdown strains solubilized in 1 N NaOH were measured (40). The optical density at 405 nm was 1.9-fold lower in the overexpression strain than in the wild type and was higher in the silenced strain (Fig. 2B). Quantitative real-time PCR (qRT-PCR) showed that the transcript levels of the predicted melanin biosynthesis genes, such as *Alb1* (*MGG_07219*), were downregulated 2.5-fold and upregulated 1.2-fold in the overexpression and silenced strains, respectively (Fig. 2C). Taken together, these results confirmed that *MoLAEA* is a negative regulator of melanin biosynthesis.

### *MoLAEA* negatively regulated sporulation and had no role in the pathogenicity of the fungus.

**FIG 3:**
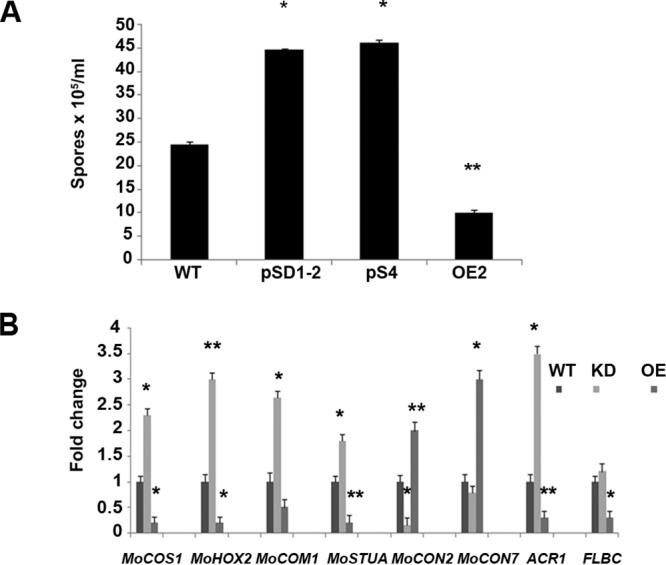
Asexual development in the *MoLAEA* overexpression and knockdown strains. (A) The spores produced by the wild-type, overexpression, and knockdown strains grown on YEG media for 10 days were collected and counted on a hemocytometer under a microscope. (B) Fold change in expression of several conidiation-related genes in the wild-type, overexpression, and knockdown strains. Error bars represent standard deviations, and asterisks represent statistically significant differences (**, *P* < 0.01; *, *P* < 0.05). KD, knockdown strain.

Secondary metabolites play important roles in fungal sporulation, and *LAEA* has also been reported to play an important role in sporulation in various filamentous fungi. To address the role played by *MoLAEA* in conidiation, we inoculated the wild-type, overexpression, and knockdown strains into yeast extract-glucose (YEG) medium and quantified the number of conidia after 10 days. The level of conidial production was significantly increased and was 1.8-fold to 1.9-fold higher in the knockdown strain whereas the level of spore production was 2.4-fold lower in the overexpression strain than in the wild type (Fig. 3A). To address how *MoLAEA* controls the sporulation process, the expression levels of eight genes involved in sporulation, namely, *MoCOS1*, *MoHOX2*, *MoCOM1*, *MoSTUA*, *MoCON2*, *MoCON7*, *ACR1*, and *FLBC*, were evaluated (41). Overexpression of *MoLAEA* reduced the expression levels of genes involved in conidiation by 2-fold to 5-fold, whereas silencing of *MoLAEA* led to 1.2-fold to 3.5-fold increases in the expression of conidiation-related genes. However, surprisingly, *MoCON2* and *MoCON7* showed increased expression (2-fold and 3-fold, respectively) in the overexpression strain and reduced expression (6.7-fold and 1.3-fold, respectively) in the silenced strain (Fig. 3B). These results confirmed that by reducing the expression of *MoCOS1*, *MoHOX2*, *MoCOM1*, *MoSTUA*, *ACR1*, and *FLBC* in M. oryzae, *MoLAEA* plays a crucial role in the development of conidiospores.

**FIG 4:**
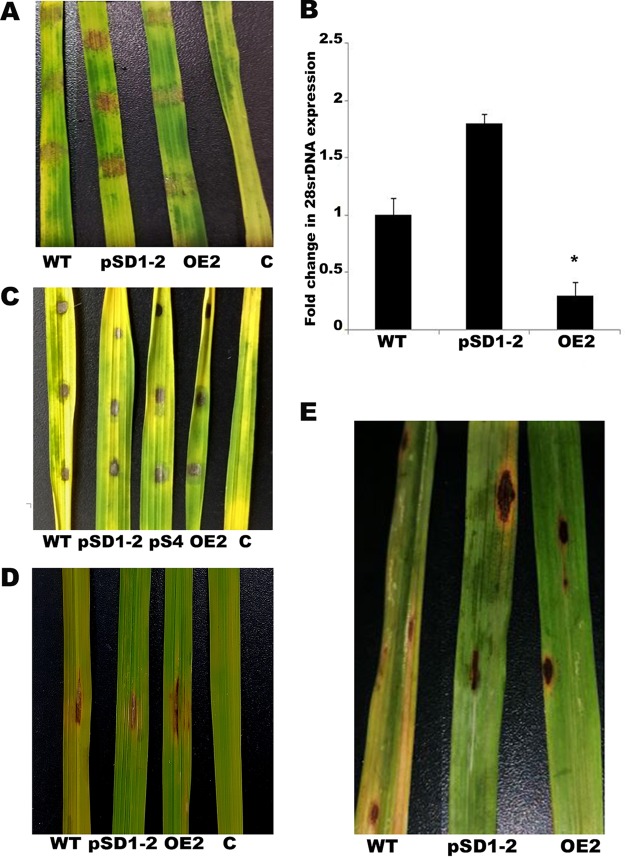
Infection assay performed with *MoLAEA* overexpression and knockdown strains. (A) Detached rice leaves (HR12) from 3-week-old rice seedlings were inoculated with hyphal plugs from wild-type, overexpression, and knockdown strains; the control (labeled “C”) contained only agar blocks. The disease symptoms were observed and photographed 5 dpi. (B) qRT-PCR analysis of the fungal biomass in the diseased rice leaves. (C) Ten-microliter volumes of spore suspensions (1 × 10^5^/ml) of the wild-type, overexpression, and knockdown strains were dropped onto detached rice leaves from 3-week-old rice seedlings, and the infected leaves were photographed 5 dpi. (D) Ten-microliter volumes of the spore suspensions were inoculated onto abraded rice leaves of 4-to-6-week-old rice seedlings. The rice plants were kept in the growth chamber at 25°C at 90% humidity for 7 days. Photographs were taken after 7 days. A gelatin solution was used for the control. (E) Five milliliters of the spore suspension (1 × 10^5^/ml) was sprayed on 3-week-old rice seedlings. The rice plants were kept in the growth chamber at 25°C at 90% humidity for 10 days. The lesion density was assessed after 10 days. The error bars represent standard deviations, and the asterisks indicate significant differences between the wild-type and mutant strains at *P* < 0.05. C, control.

To investigate the pathogenic abilities of the mycelial plugs, mycelial agar blocks from 7-day-old wild-type, overexpression, and knockdown strains were inoculated onto detached rice leaves (HR 12). The overexpression strain produced considerably reduced levels of lesions 5 days postinoculation (dpi). The level of lesion formation in the inoculated agar blocks was visibly higher in the presence of the silenced strain (Fig. 4A). To further confirm this observation and to obtain an accurate measurement of fungal biomass on rice leaves, the expression of 28S ribosomal DNA (rDNA) was checked (42) and was found to be significantly lower (3-fold) in the rice leaves inoculated with the overexpression strain and higher (1.8-fold) in the silenced strain (Fig. 4B). To further substantiate the pathogenic abilities of *MoLAEA* overexpression and knockdown strains, equal volumes of spore suspensions (1 × 10^5^/ml, 10 μl) were applied on the detached rice leaves (43). After 5 dpi, similar lesions had formed in each of the *MoLAEA* strains and the wild type (Fig. 4C). We checked the rates of conidial germination on hydrophobic surfaces, appressorium formation, and penetration and growth of invasive hyphae (IH) on rice leaf surfaces and found no significant differences between the overexpression and knockdown strains compared to the wild type (see Fig. S4). Rice leaf infection assay was performed using the spray inoculation method (44, 45) and the punch inoculation method (46, 47). Equal volumes of spore suspensions (1 × 10^5^/ml, 10 μl) were applied on slightly abraded rice leaves on plants that were 4 to 6 weeks old (46). After 7 days, it was observed that the wild-type, overexpression, and knockdown strains formed similar lesions (Fig. 4D). There were no significant differences with respect to the lesion sizes or the number of spores isolated from the lesions produced by the wild-type strain or *MoLAEA* strain on the inoculated rice plants (see Fig. S4). Leaves of 21-day-old HR12 plants were inoculated with a conidial suspension of 1 × 10^5^/ml by the use of the spray inoculation method and were observed for 10 days under conditions of high (90%) humidity at 25°C. Spindle-shaped lesions could be observed on leaves inoculated with the spores of the wild-type and *MoLAEA* strains. The development of the blast lesions was quantified, and there were no significant differences between the densities of the lesions produced by the overexpression and knockdown strains and the densities of those produced by the wild type (see Fig. S4). Taken together, these results suggest that *MoLAEA* plays no significant role in development of appressoria, penetration, or pathogenesis of M. oryzae.

### *Mo*LaeA interacted with VeA in the nucleus.

**FIG 5:**
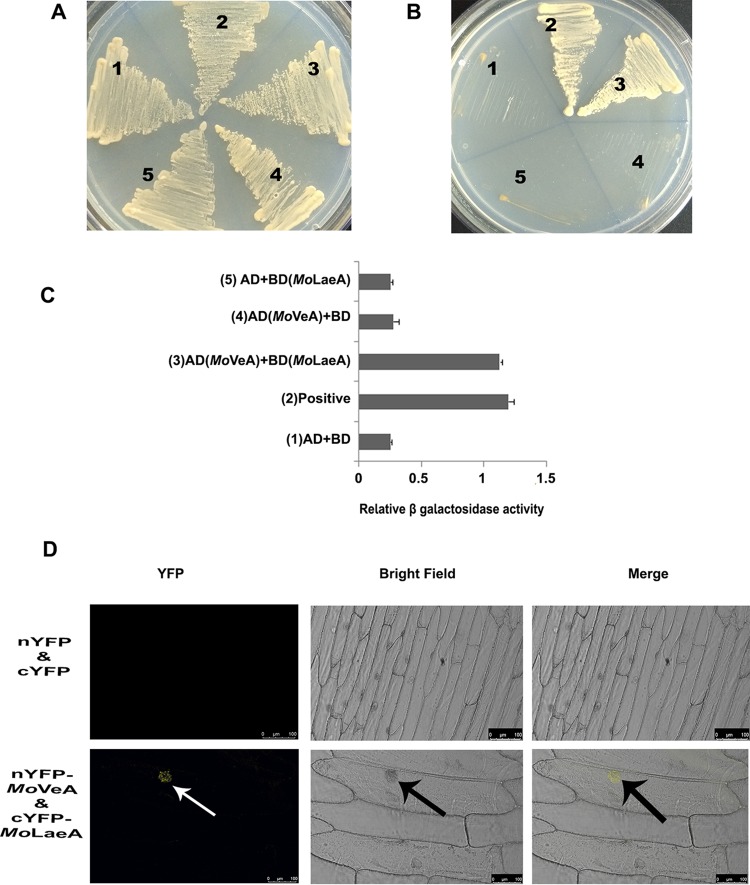
*Mo*LaeA interacts with *Mo*VeA. Yeast two-hybrid interaction between *Mo*LaeA and *Mo*VeA. (A) Growth of cotransformed yeast AH109 strain on double-dropout media. (B) Growth of cotransformed yeast AH109 strain on quadruple-dropout media. (C) Protein-protein interactions were examined using the β-galactosidase assays. The relative levels of β-galactosidase activities were calculated according to the manufacturer’s instructions (Clontech Laboratories, Inc.). The error bars represent standard deviations. Numbers 1 to 5 correspond to the constructs used. (D) BiFC assay showing that *Mo*VeA-nYFP and *Mo*LaeA-cYFP interact to form functional YFP in the nucleus. The images show transiently transformed onion epidermal cells. Scale bar, 100 μm.

LaeA formed a complex with the velvet family of proteins in A. nidulans. Therefore, it was important to ask whether *Mo*LaeA forms a complex with *Mo*VeA in M. oryzae. To examine this, we performed the yeast two-hybrid protein-protein interaction assay using the full-length coding sequences of *MoLAEA* and *MoVEA*. The growth of cotransformed yeast cells on 2D (deficient in tryptophan and leucine) and 4D (deficient in tryptophan, leucine, histidine, and adenine) was monitored in synthetically defined media. The results showed that *Mo*LaeA was able to interact with *Mo*VeA (Fig. 5A and B). Comparison of the levels of β-galactosidase activity in the *Mo*LaeA and *Mo*VeA interaction revealed that the β-galactosidase activity was approximately 3.7-fold higher than the background level and was comparable to the level seen with the positive control (4-fold higher) (Fig. 5C).

To further substantiate the interaction between *Mo*LaeA and *Mo*VeA, a BiFC (bimolecular fluorescence complementation) assay was performed. In these experiments, the full-length coding sequence of *MoLAEA* was fused to the C terminus of yellow fluorescent protein (YFP) in the pUC-SPYCE vector (*MoLAEA*-cYFP), and the full-length coding sequence of *MoVEA* was fused to the N terminus of YFP in the pUC-SPYNE vector (*MoVEA*-nYFP) (48). Interaction of *Mo*LaeA and *Mo*VeA produced YFP fluorescence in the nucleus, whereas the empty vectors did not produce any YFP fluorescence (Fig. 5D).

### *MoLAEA* was found to be involved in penicillin G biosynthesis.

**FIG 6:**
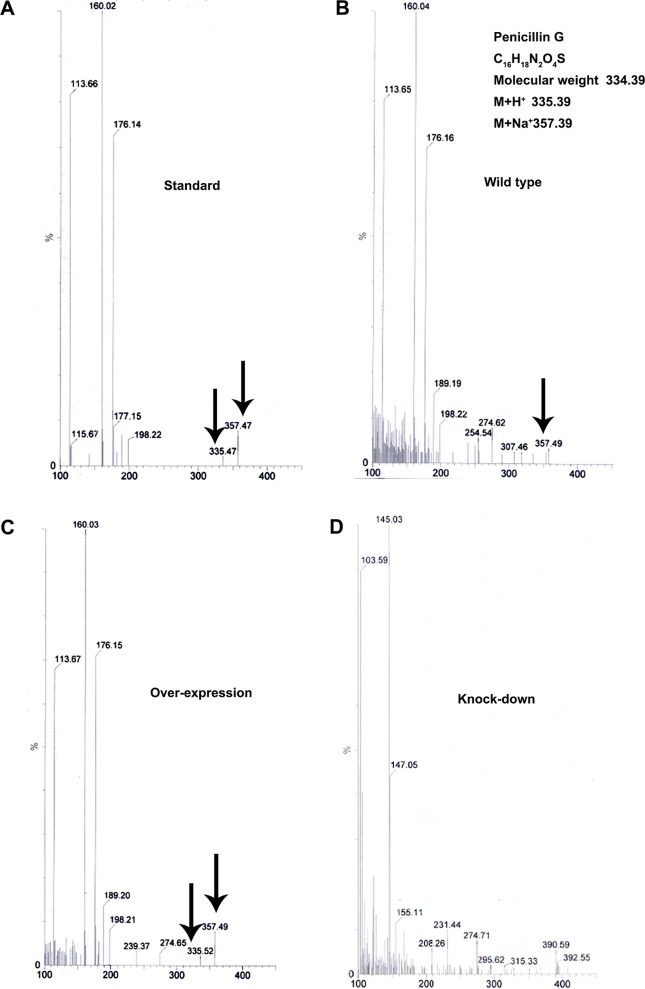
Mass spectra of the metabolites isolated from wild-type, overexpression, and knockdown strains grown in complete media at 28°C and 150 rpm for 7 days. The standard used was the Pentids 400 tablet (Abbott). (A) The mass spectra of penicillin G at 335.47 (M+H^+^) and 357.47 (M+Na^+^) in the standard. (B) Mass peak at 357.49 (M+Na^+^) in the wild type. (C) Mass peak at 335.52 (M+H^+^) and 357.49 (M+Na^+^) in the overexpression strain. (D) The knockdown strain showed a complete absence of penicillin G. The mass peaks are indicated by the arrows.

**FIG 7:**
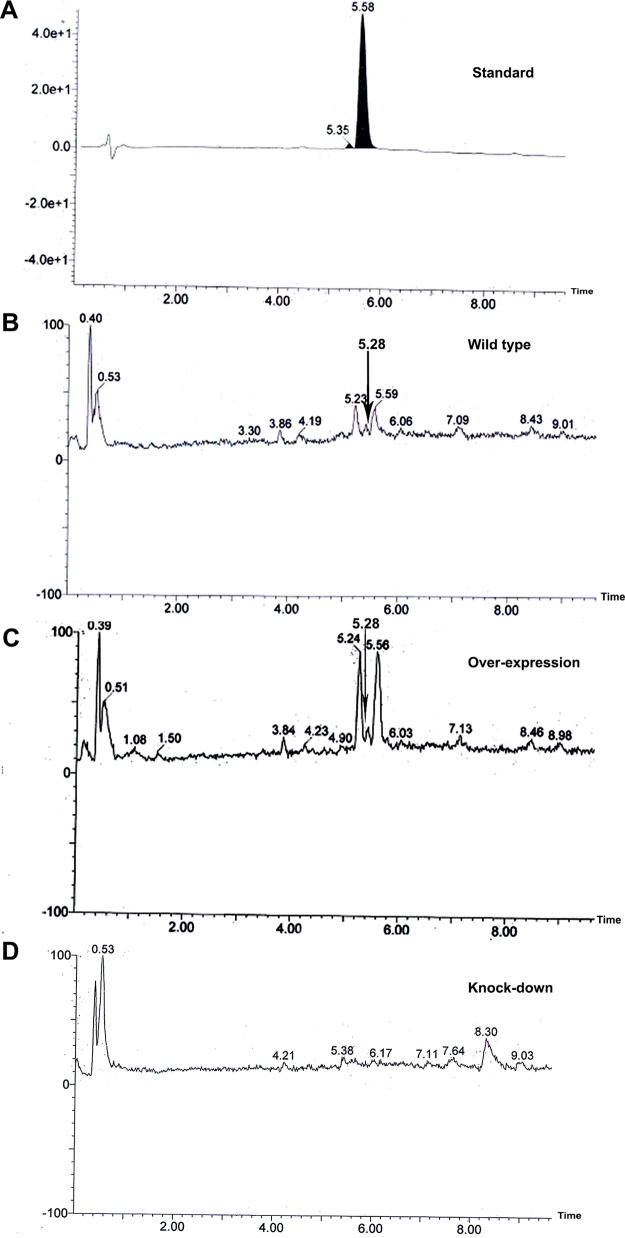
Chromatogram showing the production of penicillin G by the wild-type, overexpression, and knockdown strains. (A) The standard shows the peak at a retention time of 5.58 min. (B) The wild type shows the peak at a retention time of 5.28 min. (C) The overexpression strain shows the peak at a retention time of 5.28 min. (D) The knockdown strain shows an absence of penicillin G. The peaks are indicated by the black arrows.

To evaluate the role of *MoLAEA* in secondary metabolism, metabolite profiling was performed for the overexpression and knockdown strains, along with the wild type (unpublished data). A large number of metabolites was detected in the transformants; however, the presence of penicillin G in the wild-type and overexpression strains was striking, as its production in M. oryzae had not been reported previously. To confirm further the production of penicillin G, thin-layer chromatography (TLC) was performed. Spots developed after the contents of the chromatographic chamber were homogenized with iodine crystals at a *R_f_* value of 0.87 for the standard (Pentids 400 tablet; Abbott, Mumbai, India) and for the wild-type and overexpression strains. Notably, the spot corresponding to the positive control was absent in the silenced strain (see Fig. S5). High-performance liquid chromatography (HPLC) performed with the crude extract prepared from the mycelium of the wild-type strain and the transformants revealed that the wild-type and overexpression strains produced penicillin G whereas the knockdown strain showed complete absence of the same. Penicillin G was identified in the crude extracts according to its mass and UV/visible light (UV/VIS) absorption maxima (Fig. 6; see also Fig. 7).

**FIG 8:**
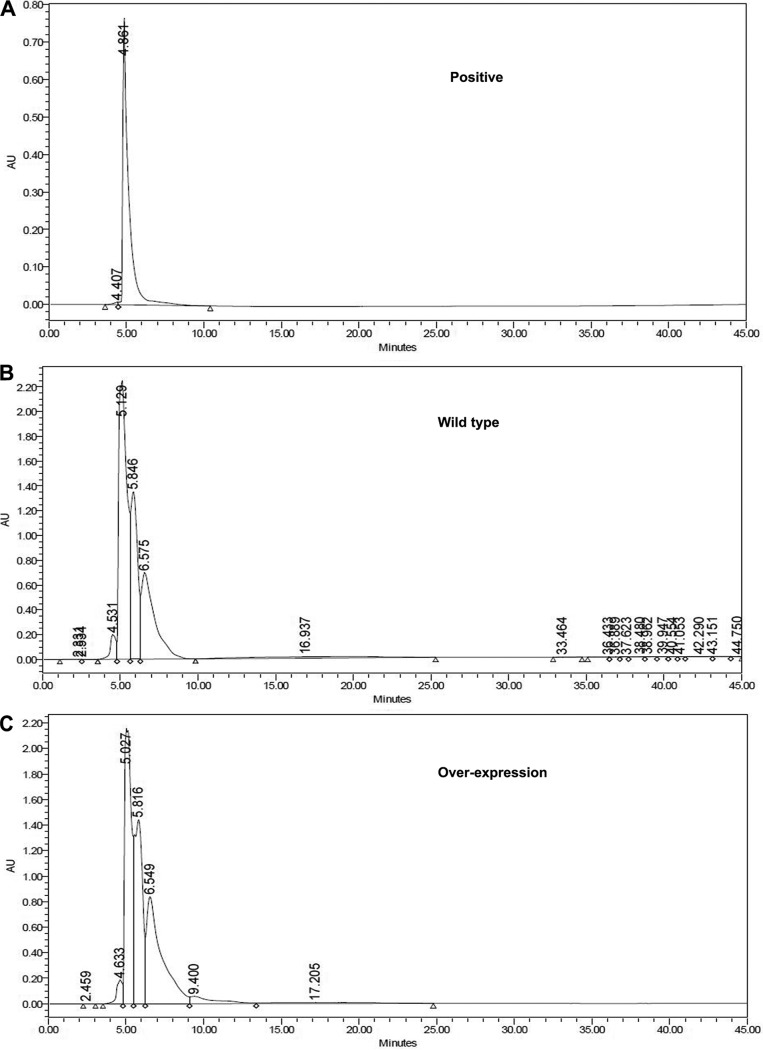
Chromatogram for checking the activity of penicillin G. (A) The standard shows the peak at a retention time of 4.86 min. (B) The wild type shows the peak at a retention time of 5.13 min. (C) The overexpression strain shows the peak at a retention time of 5.03 min. The fraction of the extract peak was collected at their respective retention times. AU, arbitrary units.

**FIG 9:**
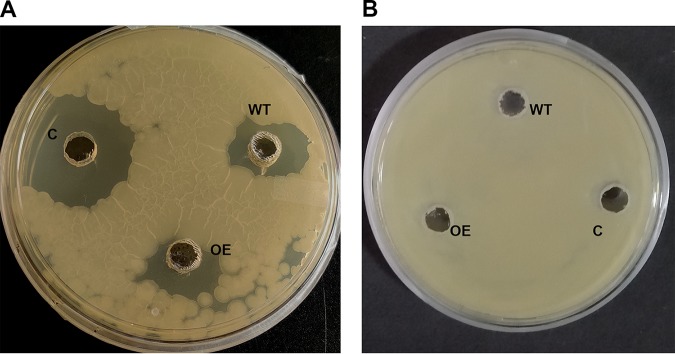
Antibiotic sensitivity of the extracted peak fraction of penicillin G. (A) One-hundred-microliter volumes of the purified fraction of the wild type (WT), overexpression (OE) strain, and positive control were tested against Bacillus subtilis. (B) One-hundred-microliter volumes of the wild type, overexpression strain, and positive control along with 6 U of β-lactamase were tested against B. subtilis. C, positive control.

Further, we carried out HPLC of crude extracts of the wild-type and overexpression strains to check the activity of the fraction corresponding to the peak in the positive control (Fig. 8).The mass of the purified fraction of the wild-type and overexpression strains was checked (see Fig. S6). The lyophilized purified fraction was dissolved in 100 μl water and tested against Bacillus subtilis to determine the activity of the purified extract peak (Fig. 9). We observed a zone of inhibition around the wild-type strain, the overexpression strain, and the positive control (Fig. 9A), whereas absolutely no zone of inhibition was seen when the same volume (100 μl) of the purified extract was incubated with 6 U β-lactamase (Fig. 9B).Taken together, our results confirmed that the molecule was benzylpenicillin produced by M. oryzae under the control of *MoLAEA.*

**FIG 10:**
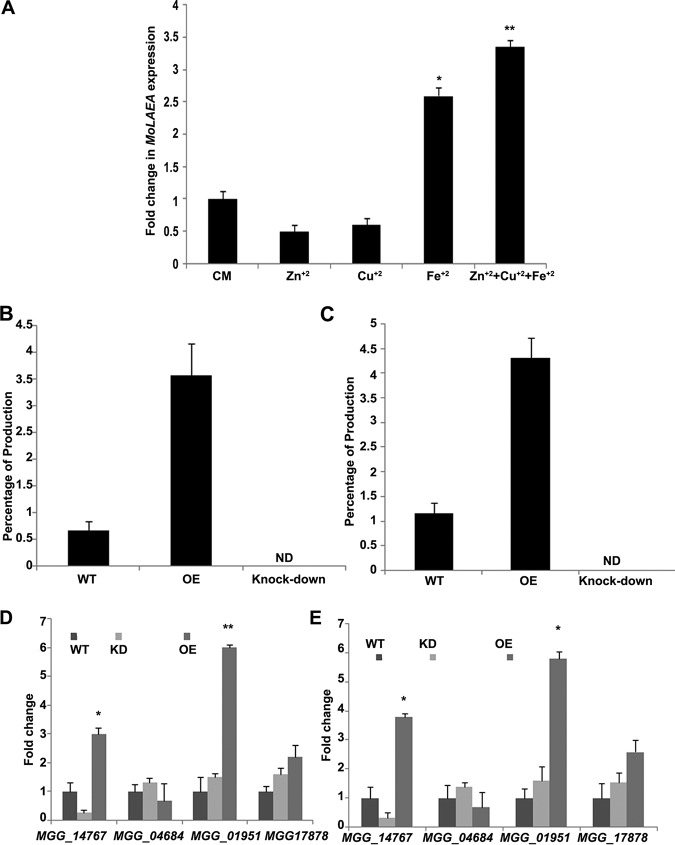
Effect of metal ions on the expression of *MoLAEA* and the genes involved in the penicillin G biosynthesis pathway. (A) The complete media were supplemented with different metal ions. The wild type was grown at 28°C and 150 rpm for 7 days. All metal ions were applied as a salt solution (5 mg/liter). (B) Quantification of penicillin G using high-performance liquid chromatography analysis of the wild-type and mutant strains 7 dpi in the absence of metal ions. (C) Quantification of penicillin G production in the presence of metal ions. (D) Fold change in expression of the homologous genes involved in the penicillin biosynthesis pathway in the absence of metal ions. (E) Fold change in expression of the homologous genes involved in the penicillin biosynthesis pathway in the presence of metal ions. Error bars represent standard deviations, and asterisks indicate statistically significant differences from the wild-type results (*, *P* < 0.05; **, *P* < 0.01). ND, not detectable; KD, knockdown strain.

To further characterize the penicillin G biosynthesis pathway in M. oryzae, the levels of expression of four genes (*MGG_14767*, *MGG_04684*, *MGG_01951*, and *MGG_17878*) were assessed in the *MoLAEA* overexpression and knockdown strains as well as in the wild type. All four genes were selected purely based on the results of a homology search of the genes involved in penicillin biosynthesis in *Aspergillus* spp. and P. chrysogenum*. MGG_14767* was upregulated by 3-fold in the overexpression strain, whereas it was downregulated 3.8-fold in the silenced strain. *MGG_04684* was upregulated 1.3-fold and downregulated 1.5-fold in the knockdown and overexpression strains, respectively.*MGG_01951* showed 1.5-fold and 6-fold increases in expression in the silenced and overexpression strains, respectively. *MGG_17878* showed 2.2-fold-higher expression in the overexpression strain and 1.6-fold-higher expression in the silenced strain. *MGG_01951* and *MGG_17878* both showed increased expression in the overexpression and knockdown strains, although the fold change level was significantly higher in the overexpression strain (Fig. 10D).

### Metal ions increased the expression of *MoLAEA* and penicillin G biosynthesis.

Studies have shown that metal ions play an important role in the expression of genes related to fungal growth, development, conidiation, and secondary-metabolite production (49). To assess the expression of *MoLAEA* in the presence of metal ions, complete media (CM) broth was supplemented with copper, iron, and zinc sulfates at the concentration of 5 mg/liter. The level of expression of *MoLAEA* was 2.6-fold higher in the presence of iron than in its absence in the wild type (not supplemented with iron). On the other hand, in the presence of copper, *MoLAEA* expression decreased by 1.7-fold. The same effect was observed with zinc, where *MoLAEA* expression decreased by 2-fold. Interestingly, when all three metal ions were added in the media, the level of expression of *MoLAEA* increased by 3.35-fold (Fig. 10A). This shows that *MoLAEA* expression was affected by the presence of metal ions, especially iron.

We further asked whether penicillin G production also increased in the presence of metal ions since their presence increased *MoLAEA* expression. We performed HPLC (see Fig. S7 and S8) and quantified the amount of penicillin G with respect to the standard both in the absence and presence of metal ions. Results showed that compared to the standard (98.45%), the wild type produced 0.67% penicillin G whereas the overexpression strain produced 3.57% of the antibiotic in the absence of metal ions (Fig. 10B). On the other hand, the wild type produced 1.17% penicillin G and the overexpression strain produced 4.32% penicillin G in the presence of metal ions (Fig. 10C). The level of expression of *MGG_14767* in the overexpression strain also increased by 3.8-fold in the presence of metal ions, while the levels of expression of the other three genes (*MGG_04684*, *MGG_01951*, and *MGG_178178*) were almost equal to those seen in the absence of metal ions (Fig. 10E).

### *MoLAEA* regulated the genes involved in secondary metabolism.

**FIG 11:**
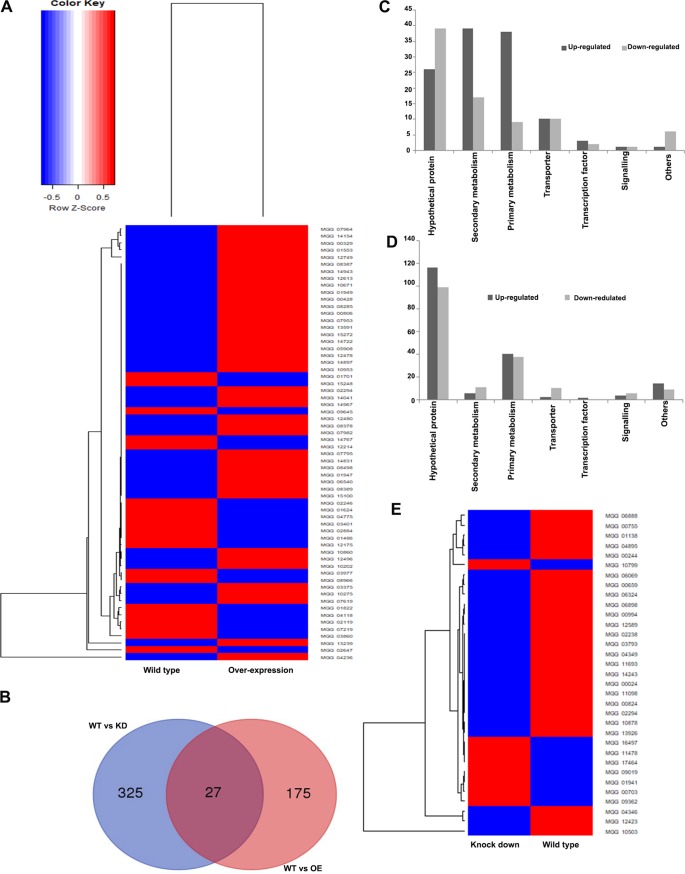
Expression profiling of the *MoLAEA* overexpression and knockdown strains. (A) Heat map representing differences between the wild-type and overexpression strains in expression levels of genes with log 2-fold changes and *P* ≤ 0.05. (B) Venn diagram of differentially expressed genes in the wild-type, overexpression, and knockdown strains. The numbers in each circle represent differentially expressed genes in each comparison, and the overlapping regions display genes that are expressed in common among the compared strains. (C) Molecular functions of the genes induced and repressed at a 2-fold expression threshold in the overexpression strain based on the Gene Ontology (GO) terms and Kyoto Encyclopedia of Genes and Genomes (KEGG) pathway analysis. (D) Molecular functions of the genes induced and repressed at a 2-fold expression threshold in the knockdown strain based on the Gene Ontology (GO) terms and Kyoto Encyclopedia of Genes and Genomes (KEGG) pathway analysis. (E) Heat map representing differences between the wild-type and knockdown strains in the expression levels of genes with log 2-fold change and *P* ≤ 0.05.

RNA sequencing (RNA-Seq) was performed to obtain more insights regarding the changes in the expression of genes associated with overexpression and silencing of *MoLAEA*. RNA from the wild-type, overexpression, and knockdown strains was isolated from 2-day-old culture grown in complete media broth at 28°C, with shaking at 150 rpm. Around 202 genes were significantly expressed in the overexpression strain, among which 118 genes were upregulated and 84 genes were downregulated (Fig. 11A). Similarly, for the silenced strain, 352 genes were found to be differentially expressed, among which 171 genes were downregulated and 181 genes were upregulated (Fig. 11E).

Compared to most other filamentous fungi, the M. oryzae genome contains a large number of genes involved in secondary metabolism (50). The expression levels of the genes involved in secondary metabolism were checked. *MoLAEA* overexpression had a greater impact than *MoLAEA* knockdown on the expression of secondary metabolism genes. Eight of the PKS genes (*MGG_12478*, *MGG_00428*, *MGG_00806*, *MGG_14831*, *MGG_13591*, *MGG_12613*, *MGG_10202*, and *MGG_08285*) were upregulated in the overexpression strain. Four of the PKS-NRPS genes (*MGG_14943*, *MGG_14897*, *MGG_15100*, and *MGG_15272*) and two NRPS genes (*MGG_14967* and MGG*_14767*) were upregulated. Furthermore, two di-terpene cyclase-encoding genes (*MGG_01949* and *MGG_14722*) and three dimethylallyl tryptophane synthase (DMATS)-encoding genes (*MGG_06540*, *MGG_10953*, and *MGG_12480*) were upregulated. *MGG_10671*, encoding a sesquiterpene synthase, was also upregulated (51–53). With the exception of *OXR1* and *CYP1*, *MoLAEA* was not found to alter the expression of genes in the *ACE1* cluster (54). The expression levels of several genes involved in primary metabolism were also altered in the overexpression and knockdown strains.The Gene Ontology (GO) and Kyoto Encyclopedia of Genes and Genomes (KEGG) pathway annotation revealed that the differentially expressed genes were involved in secondary metabolism and primary metabolism and encoded transporters, signaling pathway components, and transcription factors. Thirty-nine genes involved in secondary metabolism were upregulated in the overexpression strain (Fig. 11C), whereas 11 genes involved in secondary metabolism were downregulated in the knockdown strain (Fig. 11D). Unfortunately, most of the differentially expressed genes in the knockdown strain code for hypothetical proteins (see Excel File S1 at https://figshare.com/authors/Subhankar_Roy-Barman/8596764). The expression levels of some of the genes, namely, *MGG_04895*, *MGG_04346*, *MGG_10799*, *MGG_04826*, *MGG_00659*, and *MGG_07219*, were verified for validation of the expression profile (data not shown).

## DISCUSSION

LaeA is the global regulator of secondary metabolite biosynthesis and fungal development (5, 9, 55). Elucidating the functional role of *MoLAEA* in the regulation of secondary metabolism in M. oryzae was the main aim of this study. *MoLAEA* was identified in M. oryzae using homology searches for the *LAEA* gene from A. nidulans. It was characterized by developing overexpression and knockdown strains. We could not generate knockout mutants of *MoLAEA* even though 500 putative transformants were screened. All of the transformants were ectopic in nature. This shows that the *MoLAEA* locus has a reduced capability of homologous recombination. Studies have shown previously that the frequency of targeted gene replacement in fungi is locus dependent (56). Therefore, the low frequency of homologous recombination can be attributed to locus-specific epigenetic control, including control associated with chromatin status (57, 58). Secondary-metabolism-related genes are known to occur in clusters in the fungal genome (4). Thus, the genes in a cluster are in the same locus and are coexpressed (57). We therefore checked the expression of 15 genes upstream and downstream of *MoLAEA* in the overexpression strain. The genes in the locus encode oxygenases, reductases, hydrolases, methyltransferases, and transporters. Eight of the 15 genes showed potential involvement in secondary-metabolite synthesis. Data representing the relative fold change levels in the expression of the genes are presented in Table 1. It is evident from the fold change in expression that *MoLAEA* affects the expression of genes upstream and downstream. Neither overexpression nor silencing of *MoLAEA* affected radial growth, biomass, and cell wall integrity of M. oryzae. Similarly, A. nidulans *LAEA* knockout mutants did not show any effect on the growth of the fungus in liquid or solid media (59). Moreover, unlike primary metabolites, secondary metabolites are not directly involved in the growth and development of the fungus (5). We showed that *MoLAEA* negatively regulates melanin synthesis. Previous studies in *Aspergillus* spp. have shown that LaeA positively regulates melanin synthesis as well as the expression of *Alb1* (16, 60). However, in Aspergillus fumisynnematus and Cochliobolus heterostrophus, overexpression of *LAEA* led to decreased pigment production (21, 61). Similarly, in the case of *MoLAEA*, overexpression led to a 2.5-fold reduction in expression of *Alb1*. *MoLAEA* also acts as the negative regulator of sporulation, which is similar to what is observed in A. fumisynnematus and C. heterostrophus (21, 61). However, in A. flavus, A. carbonarius, A. alternata, P. chrysogenum, T. atroviride, and D. septosporum, LaeA acts as a positive regulator of sporulation (26, 27, 32, 62–64). The conidiation-related genes, except *MoCON2* and *MoCON7*, showed reduced expression in the overexpression strain and higher expression in the silenced strain. *MoCON2* and *MoCON7* expression increased (2-fold and 3-fold, respectively) in the overexpression strain and decreased (6.7-fold and 1.3-fold, respectively) in the silenced strain. The reason for the increased expression of *MoCON2* and *MoCON7* is not clear. One of the reasons might be that they are not directly involved in sporulation. The *MoCON7* mutants formed normal spores, although the spores did not form appressoria. On the other hand, the spores of *MoCON2* mutants were previously shown to be morphologically deformed and to have a reduced ability to form appressoria (65). *MoLAEA* was found not to be involved in pathogenesis in spite of its role in secondary metabolism. One possible explanation which further needs investigation is that *MoLAEA* might be involved in the long-term role of helping the fungus to adapt itself to the continuously changing environmental conditions. However, inoculation of the mycelial plug onto detached rice leaves resulted in lower levels of lesion in the overexpression strain. This might have been due to the fact that overexpression strain produced fewer spores, which led to lower levels of lesion formation. Further, under conditions of inoculation with equal numbers of spores, the overexpression and knockdown strains produced lesions similar to those seen with the wild type.

**TABLE 1:** Expression of the genes upstream and downstream to *MoLAEA* in the overexpression strain

Gene	Predicted function	Fold change
*MGG_07927*	Endochitinase 1	10
*MGG_07937*	2-Dehydropantoate 2-reductase	4
*MGG_07953*	NADPH-P450 reductase	4
*MGG_07954*	Epioxide hydrolase 2	12
*MGG_07957*	Uridine permease	6
*MGG_07958*	2-Oxoglutarate 3-dioxygenase	2
*MGG_07962*	Hypothetical protein	2
*MGG_07965*	Alkaline proteinase	1.7
*MGG_13927*	Hypothetical protein	3
*MGG_07968*	High-affinity nicotinic acid transporter	1.4
*MGG_07969*	Hypothetical protein	2
*MGG_07970*	Hypothetical protein	1.6
*MGG_07982*	Cytochrome P450 monooxygenase	6
*MGG_10852*	Methionine synthase	4
*MGG_10860*	Sterol 24-C-methyltransferase	9

The velvet family proteins VosA, VelB, VelC, and VeA are involved in morphogenesis and regulation of secondary metabolites in fungi (24, 66). LaeA forms a complex with two members of the velvet family, VelB and VeA, and participates in fungal development and secondary-metabolite synthesis (23). The complex is formed in the nucleus, as VeA is translocated to the nucleus in dark (67). *Mo*LaeA was found to form a complex with *Mo*VeA in the nucleus, which represents the first report of such an interaction in M. oryzae. Thus, we can conclude that the mechanism via which LaeA and the velvet proteins function is conserved among filamentous fungi.

One of the striking phenotypes of the overexpression strain was the increased production of penicillin G compared to that produced by the wild type. To confirm whether the molecule was indeed benzylpenicillin, the fraction with the extracted peak collected for the wild type and the overexpression strain was tested against B. subtilis. A zone of inhibition was observed around the wild type, the overexpression strain, and the positive control, but when the same fraction was incubated with 6 U of β-lactamase, no zone of inhibition was observed. Expression of some of the genes homologous to the genes involved in penicillin biosynthesis in *Aspergillus* spp. and *Penicillium* spp. has been studied. The first step in the penicillin biosynthesis pathway is the formation of the *d*-(l-*a*-aminoadipyl)-l-cysteinyl-d-valine (ACV) tripeptide. This rate-limiting reaction is catalyzed by a single enzyme, *d*-(l-*a*-aminoadipyl)-l-cysteine-d-valine synthetase (ACVS), encoded by the *acvA* (pcbAB) gene. The ACV tripeptide is formed from its amino acid precursors via a nonribosomal enzyme thiotemplate mechanism (68–72). When we searched for homologous genes in M. oryzae, *MGG_14767* was the best candidate gene, with 32.3% similarity. Overexpression of a phenylacetyl-coenzyme A (CoA) ligase gene (*phl*) in P. chrysogenum leads to a 35% increase in penicillin production. *MGG_01951*, a homolog which is 48.2% similar to *phl*, has been reported previously in M. oryzae (73). It is known that NRPS and PKS require posttranslational phosphopantetheinylation by a PPTase (4’-phosphopantetheinyl transferase) to become active. Amplification of *ppt* increases isopenicillin N and benzylpenicillin biosynthesis. MGCH7_ch7g840 (*MGG_17878*) from M. oryzae (EnsemblFungi database accession number XP_001522742) is homologous to *ppt* of P. chrysogenum (74). *PhacA* encodes P450 monooxygenase, which catalyzes phenylacetate 2-hydroxylation. It degrades phenylacetate to fumarate and acetoacetate. Disruption of *PhacA* results in penicillin overproduction (75). *MGG_04684* shows 74% homology to the A. nidulans *PhacA* gene. *MGG_14767* is 3-fold upregulated in the overexpression strain, whereas it is 3.8-fold downregulated in the knockdown strain. The fact that the overexpression strain produces more penicillin G than the knockdown strain might be due to the fold change differences in the levels of expression of *MGG_14767*, which may act as the deciding factor or as an important gene governing the penicillin biosynthesis pathway, although it is characteristically different from *acvA*.

**FIG 12:**
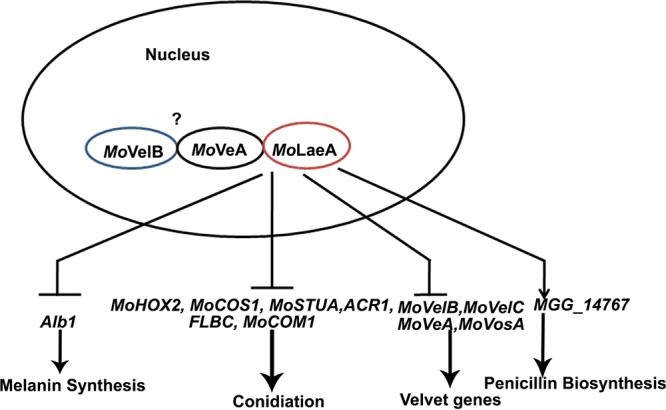
Model showing the functional role of *MoLAEA* in M. oryzae.

Taken the data together, *MoLAEA* was found to regulate secondary metabolism in M. oryzae and to be involved in penicillin G biosynthesis. It negatively regulated melanin synthesis and sporulation (Fig. 12). Further studies will broaden our understanding regarding the mechanism of *MoLAEA* action and will assist in identifying new metabolites that are under the control of *MoLAEA*. This is especially important, as a large number of bioactive natural compounds in M. oryzae are apparently still unknown. Future research should focus on the biosynthesis of penicillin G and on understanding the genetic regulation of secondary metabolism in M. oryzae.

## MATERIALS AND METHODS

### Strains and growth conditions.

M. oryzae isolate B157 was used as the wild-type strain in the present study. B157 was isolated during 1990s from Maruteru, near Hyderabad, India (76, 77). A pure culture of M. oryzae was obtained from single spore and was used for all the experiments carried out in this study. The wild-type, overexpression, and knockdown strains were maintained on yeast extract-glucose media (yeast extract 2%, dextrose 1%, and agar 2%) at 28°C. For the growth assay, the wild-type and mutant strains were grown on complete agar media (glucose 1%, peptone 0.5%, casein acid hydrolysate 0.1%, yeast extract 0.1%, sodium nitrate 0.6%, potassium chloride 0.05%, magnesium sulfate 0.05%, and potassium dihydrogen phosphate 0.15%, pH 6.5) for 10 days at 28°C. Liquid complete medium was used to prepare mycelia for genomic DNA extraction (78) and for RNA isolation (RNeasy plant minikit; Qiagen, India).

### Identification of *MoLAEA* and generation of *MoLAEA* overexpression and knockdown strains.

The A. nidulans LaeA sequence was used as the query sequence against the M. oryzae database (BroadMIT version 6). *MGG_07964* was considered the best hit, with 38% similarity at the protein level. Primers were designed that covered some portions of the methyltransferase domain and the C-terminal region. All the primers used in the study are listed in Table S1 at https://figshare.com/authors/Subhankar_Roy-Barman/8596764. A 396-bp portion was amplified using the coding sequence of the wild type as the template. The silencing construct was developed in the pSD1 (psilentDual1) vector containing Geneticin as the selection marker and dual *pgpd* and *pTrpC* promoters in either orientation. A 396-bp gene fragment was amplified using *Pfu* polymerase (NEB, USA) and was cloned into XbaI and HindIII sites of the vector. Alternatively, a silencing construct was also generated in pSilent1. The 386-bp gene fragment was cloned in sense and antisense orientations on either side of the intronic region (cutinase intron) in the vector such that it formed a hairpin. The sense strand was cloned in the XhoI and HindIII sites, whereas the antisense strand was cloned in the BglII and KpnI sites of the pSilent1 vector. An overexpression construct was made in the pSilent1 vector containing *Hpt*, or the hygromycin phosphotransferase gene, as the selection marker by removing the intronic region in the vector and cloning the gene at the HindIII and KpnI sites, placed under the control of the *pTrpC* constitutive promoter. The plasmid constructs were transformed into M. oryzae via protoplast transformation. CM broth (40 ml) was inoculated, and the culture was grown for 2 days at 28°C and 150 rpm. The fungal balls were filtered by the use of Miracloth (Calbiochem, San Diego, CA, USA) and were washed with sterile distilled water followed by resuspension in 40 ml of 1 M sorbitol containing lysing enzyme from Trichoderma harzianum (Sigma, St. Louis, MO, USA) at a concentration of 1 mg/ml. This was incubated overnight at 28°C at 100 rpm for protoplasting. The protoplasts were filtered the next day, washed, and then resuspended in 200 μl STC buffer (1 M sorbitol, 50 mM Tris/HCl [pH 7.4], 50 mM calcium chloride) containing 5 μg plasmid DNA. After incubation for 15 min at 4°C, 1 ml PTC buffer (40% [wt/vol] polyethylene glycol 3550 [Sigma-Aldrich], 50 mM Tris-chloride [pH 7.4], 50 mM calcium chloride) was added and the mixture was incubated at 28°C for 30 min. A 3-ml volume of CMS (CM with 1 M sorbitol) was added to this mixture, followed by incubation for 16 h at 28°C with shaking at 100 rpm. On the next day, 5 ml of molten regeneration media (CM with 0.4% agarose) was added and the mixture was plated on selection plates containing 200 μg/ml hygromycin B (InvivoGen, San Diego, CA, USA) for the pSilent1 vector or 250 μg/ml Geneticin (TCI, Tokyo, Japan) for the pSD1 vector. After three successive selections performed on the respective selection media, the presence of the transgene was confirmed using PCR. Southern blotting was performed as described previously (79). Ten micrograms of genomic DNA was digested overnight with HindIII for the silencing transformants and with XbaI for the overexpression transformants. The digested product was separated by the use of 0.7% agarose gel electrophoresis at 40 V for 6 h. The capillary blot transfer was performed for 16 to 18 h onto a positively charged nylon membrane (Hybond N+, Amersham, Buckinghamshire, United Kingdom).A AlkPhos direct labeling and detection system with CDP-Star (Amersham, GE Healthcare, Buckinghamshire, United Kingdom) was used to label the probes. *PtrpC* was used as the probe. Hybridization and chemiluminescent detection were performed according to the manufacturer’s instructions.

### Real-time PCR analysis.

The total RNA of the transformants and the wild type was isolated from the mycelia of 2-day-old CM broth using a Qiagen RNeasy plant minikit (Qiagen, New Delhi, India) per the manufacturer’s instructions. Genomic DNA contamination was removed using RNase-free DNase (Qiagen). The integrity was checked on a 1% agarose gel, and the RNA was quantified using a Bio-Rad spectrophotometer. First-strand cDNA was synthesized using a Thermo Scientific RevertAid H minus first-strand cDNA synthesis kit. qRT-PCR was performed on an Applied Biosystems Step One real-time PCR machine using Power SYBR green PCR master mix (Applied Biosystems, Woolston, United Kingdom). The relative amounts of the target gene transcripts in terms of fold change were calculated using the expression 2^–ΔΔ^*^CT^*, where “*CT*” represents “threshold cycle” and “ΔΔ*CT*” represents (C*T*_gene of interest_ – *CT*_actin_)_test condition_ – (*CT*_gene of interest_ – *CT*_actin_)_control_. Each real‐time PCR was performed in triplicate, and values for each gene were normalized to the expression level of the wild‐type B157 isolate.

### Hyphal growth and dry weight determination.

Small agar blocks were cut from a 7-day-old culture plate and placed on the center of the CM plate. The plates were incubated at 28°C, and growth was checked on days 5, 7, and 10. Photographs were taken on day 10. Dry weight was determined by growing the fungus for 2 days in CM broth. The mycelia were filtered using Whatman filter paper number 1. The filter paper, along with the fungal filtrate, was incubated at 50°C for 48 to 72 h (49). Here, the control was the filter paper without the mycelium; its weight was measured first followed by measurement of the weight of the filter paper with the mycelium.

### Melanin assay.

The wild-type, overexpression, and knockdown strains were grown in complete medium broth at 28°C and 150 rpm for 2 days. A 100-milligram volume of the biomass was crushed in liquid nitrogen and solubilized in 1 N NaOH for 1 h at 80°C, followed by centrifugation at 12,000 × *g* for 10 min. The absorbance of the supernatant was measured at 405 nm (40).

### Sporulation and infection assay with rice leaves.

Conidia were isolated by scraping the mycelia from 10-day-old YEG cultures, followed by filtration through Miracloth (Calbiochem). The spores were suspended in 10 μl autoclaved distilled water and counted using a hemocytometer. Pathogenicity assay was performed using leaves of 3-week-old rice seedlings (Oryza sativa cultivar HR12). The leaves were cut into smaller (2-to-3-cm-square) pieces and were placed in 2% agar. Five microliters of spore suspensions with equal numbers of spores (1 × 10^5^ spores/ml) were inoculated on the detached rice leaves (43). Hyphal plugs of the wild type as well as the transformants were inoculated on the detached rice leaves. The plates were incubated at 28°C for 5 days under moist conditions. Photographs were taken after 5 days. The experiments were repeated three times. The levels of expression of the 28S rDNA were checked for the growth of the fungal biomass on the rice leaves (42). The whole-plant infection assay was performed using the punch inoculation and spray inoculation method. Spores were isolated from 10-day-old plates of the wild-type, overexpression, and knockdown strains. Ten microliters of the spore suspension (1 × 10^5^/ml) was applied to slightly punctured sites on rice leaves that were 4 to 6 weeks old, and the sites were then covered with adhesive tape (46, 47). The inoculated plants were incubated in a growth chamber at 25°C and 90% humidity and in the dark for the first 24 h, followed by a 12-h/12-h light/dark cycle. Photographs were taken 7 days postinoculation. Furthermore, to measure the sporulation rate, the infected parts, including the lesions, were immersed in 100 μl distilled water–1% Tween 20. The samples were subjected to vigorous vortex mixing for 2 min to dislodge the spores, and the spores were counted using a hemocytometer (46). For spray inoculation, 5-milliliter volumes of conidial suspensions (1 × 10^5^/ml) were sprayed onto 21-day-old rice leaves with a sprayer. Inoculated plants were incubated in a growth chamber at 25°C at 90% humidity in the dark for the first 24 h, followed by a 12-h/12-h light/dark cycle. The photographs were taken 10 days postinoculation. Disease lesion densities were recorded from 20 infected leaves using a 5-cm-square section of each leaf (80). Mean size of lesions was measured using Image J software (https://imagej.nih.gov/ij/) (81).

The conidial germination rate was determined as the mean percentage of conidia that had germinated on hydrophobic surfaces after 24 h. Three replicates of 50 conidia were counted for each observation. Rates of appressorium formation and penetration were determined by analyzing 50 spores or appressoria per rice cuticle after 24 h. This was repeated three times to determine the mean value (82, 83). *In planta* biotrophic growth was determined using a 4-point scale where a score of 1 refers to IH length of less than 10 mm with no branching, a score of 2 refers to IH length of 10 to 20 mm with 0 to 2 branches, a score of 3 refers to IH length of more than 20 mm and/or with more than 2 branches within one cell, and a score of 4 refers to IH that had spread to adjacent cells (82–84)

### Liquid chromatography-mass spectrometry (LC/MS) and thin-layer chromatography.

The wild-type, overexpression, and knockdown strains were grown in the CM broth at 28°C and 150 rpm for 7 days. Phenylacetic acid (Sigma-Aldrich) was added to the culture media at a concentration of 0.2 g/liter after 48 h. One hundred milligrams of the biomass was ground to fine powder using liquid nitrogen and a mortar and pestle. To the powdered sample, 2 ml extraction buffer (water, methanol, and acetonitrile [1:2:2]) was added. The samples were subjected to vortex mixing for 1 min, followed by sonication for 10 min in an ultrasonic water bath. The samples were centrifuged at 12,000 × *g* for 15 min at 4°C, and the supernatants were transferred to fresh tubes. Ten-microliter volumes of the supernatants were injected into an HPLC system (Alliance Waters 2695) (85). The solvent used was acetonitrile–water–0.1% formic acid. The column used was a reverse-phase C_18_ column. The detection wavelength range was 210 to 400 nm. A Pentids 400 penicillin G potassium tablet (Abbott) was used as the standard. Furthermore, 2-μl samples were placed on the silica gel using a Hamilton microsyringe (Hamilton, USA). The mobile phase consisted of ethyl acetate, water, and acetic acid (60:20:20). The chromatographic chambers were saturated with the mobile phase for 30 min. The plates were developed over a distance of 15 cm, followed by drying in hot air. The spots were then visualized by placing the plates in a chromatographic chamber saturated with iodine vapors (86).

### Bioassay using the purified peaks of penicillin G.

The method of isolation of the fungal extract was as mentioned above. Two-milliliter volumes of crude extract of the wild-type and overexpression strains were injected in an HPLC system (Waters 2998). A 1-ml volume of the positive control dissolved in methanol was injected. The crude extract, as well as the positive control, were subjected to filter sterilization before injection to remove all particulate matter that might cause a problem during the flow. The flow rate was maintained at 4 ml/min. Solvent A consisted of HPLC-grade water and 0.1% formic acid, and solvent B was acetonitrile. The detection wavelength was 220 nm, and the run time was 45 min. The fraction corresponding to the peak in the positive control was collected for the wild type and the overexpression strain. The mass of the collected fraction was checked using a Xevo G2-XS QToF (quadrupole time of flight) mass spectrometer. The blank used contained 500 μl methanol and 500 μl water. The positive control contained 480 μl methanol, 480 μl water, and 40 μl purified fraction. The wild-type sample consisted of 450 μl methanol, 450 μl water, and 100 μl purified fraction. The overexpression strain sample consisted of 475 μl methanol, 475 μl water, and 50 μl purified fraction. The flow rate was maintained at 5 μl/min.

To perform the antibiotic sensitivity test using the purified extract, the solvent was evaporated at 40°C and lyophilized at –48°C and finally dissolved in 100 μl distilled water. Subsequently, 100 μl sample with or without 6 U β-lactamase (MP Biomedicals, OH, USA) was added to the wells in Luria-Bertani plates spread with B. subtilis culture (16). The plates were incubated overnight at 37°C. The photograph of the zone of inhibition was captured the next day.

### RNA sequencing.

Total RNA was isolated from the wild-type, overexpression, and knockdown strains by the use of a Qiagen RNeasy Plant minikit according to the manufacturer’s instructions. The quality and quantity of the extracted RNA were assessed using Qubit (a double-stranded RNA high-sensitivity [dsRNA HS] kit from Invitrogen) and a TapeStation system (RNA screen tape; Agilent). The extracted RNA met the Illumina standards and was used for library preparation. RNA was prepared from two biological replicates and used for independent library preparations. The library was prepared using TruSeq RNA Library Prep Kit v2 from Illumina and an Illumina standardized protocol. The prepared libraries were quantified on Qubit using the dsDNA HS kit and validated on the TapeStation system using D1000 screen tape and for quantification using real-time PCR (Kapa library quantification kit). The prepared libraries met the Illumina standards and were processed further for sequencing. The library was denatured using NaOH followed by neutralization using 0.2 N Tris (pH 7). The final library was loaded onto a NextSeq 500 reagent cartridge for cluster generation and sequencing. The genome sequence of M. oryzae strain 70-15 was used as the reference genome and was downloaded from NCBI. Quality-processed Illumina paired-end reads were used for reference-based alignment performed with Tophat (version: v2.1.0) and in-house tools (87). The reference-based aligned reads from Tophat (version: v2.1.0) was used for identification of transcripts with Cufflinks (version: v2.2.1) (88). Cufflinks assembled the individual transcripts from RNA-Seq reads that were aligned to the reference genome of M. oryzae strain 70-15. Differential gene expression analysis was performed using Cuffdiff (0.1). The differentially expressed genes were filtered based on log2 fold change and a *P* value of ≤0.05. Heat maps of the genes whose expression changes were statistically significant were generated using an in-house R-script. The gene ontology analysis was performed with differentially expressed genes with the parameter of log2 fold change and a *P* value of ≤0.05.

### Yeast two-hybrid and BiFC assays.

The yeast two-hybrid assay was performed using a Matchmaker GAL4-based two-hybrid system as recommended by Clontech Laboratories, Inc. (WI, USA). To investigate the protein-protein interaction, full-length *MGG_08556* (*MoVEA*) was cloned into the EcoRI-BamHI restriction sites of the pGADT7 vector (Clontech Laboratories, Inc.) to produce translational fusion proteins with the activation domain. Full-length *MGG_07964* (*MoLAEA*) was cloned in the pGBKT7 vector (Clontech Laboratories, Inc.) in EcoRI-BamHI restriction sites to generate a translational fusion with the binding domain. To assess protein-protein interactions, the corresponding plasmids were cotransformed into yeast strain AH109 according to the manufacturer’s instructions. Successfully transformed colonies were identified on quadruple-dropout media lacking Trp, Leu, His, and Ade. The protein-protein interactions were also examined using β-galactosidase assays with chlorophenol red-β-d-galactopyranoside as the substrate. The relative levels of β-galactosidase activity were calculated according to the manufacturer’s instructions.

For the BiFC assay, the full-length coding sequences of *MoLAEA* (*MGG_07964*) and *MoVEA* (*MGG_08556*) were cloned in the XbaI and KpnI sites of pUC-SPYCE and pUC-SPYNE, respectively (48). The constructs were transformed in *Agrobacterium* sp. strain GV3101, followed by transformation in living onion epidermis (89). The onions injected with the required combinations of the constructed vectors were incubated in the dark for 48 h at 28°C for expression of the transfected DNA and reconstruction of functional YFP. The onion epidermis was mounted on glass slides and observed under a confocal microscope (Leica TCS SP8) with a standard filter set.
